# Fungal Histidine Phosphotransferase Plays a Crucial Role in Photomorphogenesis and Pathogenesis in *Magnaporthe oryzae*

**DOI:** 10.3389/fchem.2017.00031

**Published:** 2017-05-19

**Authors:** Varsha C. Mohanan, Pinal M. Chandarana, Bharat. B. Chattoo, Rajesh N. Patkar, Johannes Manjrekar

**Affiliations:** ^1^Bharat Chattoo Genome Research Centre, Department of Microbiology and Biotechnology Centre, Maharaja Sayajirao University of BarodaVadodara, India; ^2^Department of Microbiology and Biotechnology Centre, Maharaja Sayajirao University of BarodaVadodara, India

**Keywords:** two-component system, His-Asp phosphorelay, *Magnaporthe oryzae*, conidiation, light response, host invasion, rice blast

## Abstract

Two-component signal transduction (TCST) pathways play crucial roles in many cellular functions such as stress responses, biofilm formation, and sporulation. The histidine phosphotransferase (HPt), which is an intermediate phosphotransfer protein in a two-component system, transfers a phosphate group to a phosphorylatable aspartate residue in the target protein(s), and up-regulates stress-activated MAP kinase cascades. Most fungal genomes carry a single copy of the gene coding for HPt, which are potential antifungal targets. However, unlike the histidine kinases (HK) or the downstream response regulators (RR) in two-component system, the HPts have not been well-studied in phytopathogenic fungi. In this study, we investigated the role of HPt in the model rice-blast fungal pathogen *Magnaporthe oryzae*. We found that in *M. oryzae* an additional isoform of the *HPT* gene *YPD1* was expressed specifically in response to light. Further, the expression of light-regulated genes such as those encoding envoy and blue-light-harvesting protein, and PAS domain containing HKs was significantly reduced upon down-regulation of *YPD1* in *M. oryzae*. Importantly, down-regulation of *YPD1* led to a significant decrease in the ability to penetrate the host cuticle and in light-dependent conidiation in *M. oryzae*. Thus, our results indicate that Ypd1 plays an important role in asexual development and host invasion, and suggest that *YPD1* isoforms likely have distinct roles to play in the rice-blast pathogen *M. oryzae*.

## Introduction

A typical two-component signaling pathway involves a transmembrane histidine kinase (HK), which senses distinct external signal(s)/cue(s) and a response regulator (RR) that activates the downstream mitogen-activated protein (MAP) kinase cascade or directly regulates transcription of the target genes. Multiple components or steps are involved in a more complex two-component signal transduction (TCST) pathway, which may consist of hybrid proteins with both kinase and receiver domains, more than two proteins in the pathway or multiple His-Asp phosphotransfer events (Alex and Simon, 1994; Stock et al., 2000). The histidine phosphotransfer (HPt) domain in a hybrid HK or in an independent protein is an important component of the His-Asp phosphorelay system, in which it mediates the phosphotransfer between the upstream sensor HK and downstream RR. Although the TCST pathways are largely conserved across the species, they can carry out diverse functions, likely due to the expanded family of HKs.

While TCST pathways are very common in bacteria, they do occur, albeit less commonly, in plants and lower eukaryotes such as fungi and slime molds. TCST pathways in fungi have been found to be involved in fungicide sensitivity, responses against oxidative and osmotic stresses, secondary metabolism, morphogenesis, cell wall integrity, and asexual and sexual development (Li et al., 2010). In the filamentous fungus *Aspergillus nidulans*, the RRs SrrA and SskA have been reported to be involved in asexual development and conidiospore viability (Vargas-Perez et al., 2007). Similarly, *Botrytis cinerea* HK Bos1 has a function in spore formation (Liu et al., 2008). While *Aspergillus fumigatus* HK Fos1 is required for conidiophore development, another HK, TscB, was found to be dispensable for sporulation but important for osmo-sensing and regulation (Pott et al., 2000; Furukawa et al., 2002; Du et al., 2006). In *Fusarium graminearum*, the HK FgOs1 is involved in regulation of secondary metabolism (Ochiai et al., 2007). In addition to functions in growth and development, TCST pathways are vital determinants of pathogenicity in animal and plant fungal pathogens, such as *Candida albicans, Cryptococcus neoformans, A. fumigatus, Cochliobolus heterotrophus, Gibberella zeae, Fusarium oxysporum, B. cinerea*, and *Alternaria brassicicola* (Alex et al., 1998; Clemons et al., 2002; Bahn et al., 2006; Viaud et al., 2006; Cho et al., 2009; Oide et al., 2010; Rispail and Di Pietro, 2010). Genome of the rice-blast fungal pathogen *Magnaporthe oryzae* has ten HKs, one HPt and three RRs. Like in other fungi, HKs and RRs in *M. oryzae* have been shown to function in pathways involved in various stress responses, morphogenesis, growth and development (Motoyama et al., 2005a,b, 2008; Zhang et al., 2010; Jacob et al., 2014). For example, HKs Hik5 and Hik8 are reported to be important for conidiation and conidial morphology in *M. oryzae* (Jacob et al., 2014).

While plants such as *Arabidopsis thaliana* and *Oryza sativa* have more than one HPt, most fungi have a single copy gene. *Saccharomyces cerevisiae* Ypd1 (ScYpd1), the first fungal HPt identified, is essential for viability (Posas et al., 1996). Similarly, HPt is essential for viability in *C. neoformans* or filamentous fungi like *Neurospora crassa* and *A. nidulans* (Banno et al., 2007; Vargas-Perez et al., 2007; Lee et al., 2011). On the other hand, in *Schizosaccharomyces pombe* or *C. albicans* HPt is dispensable for viability (Aoyama et al., 2000; Mavrianos et al., 2014). While the HPt domain is highly conserved, a small N-terminal stretch is variable in fungi. Interestingly, the N-terminal extension of Mpr1 is important for the interaction between the HPt and its downstream RR in *S. pombe* (Tan et al., 2007).

HPts are suitable antifungal targets due to their absence in animal systems. However, most studies to date have focused on HKs, and HPts have not been well-characterized in plant pathogenic fungi thus far. In *M. oryzae*, deletion of the *HPT* gene (Mo*YPD1*) did not affect viability; however, absence of the MoYpd1 function led to a complete loss of conidiation (Jacob et al., 2015). *In vitro* assays with the vegetative cultures of the Δ*ypd1* strain showed altered sensitivity of the mutant toward osmotic stress and antifungal treatment (Jacob et al., 2015).

Here, we studied the expression pattern of the *HPT* gene (hereafter called *YPD1*) in response to light, in *M. oryzae*. Using a gene silencing approach, we functionally characterized *YPD1*, wherein asexual development (which is largely dependent on light) and pathogenesis were studied upon down-regulation of the *HPT* gene in *M. oryzae*. We show that Ypd1 plays a critical role in conidiation, likely via regulation of light-responsive gene expression, and in proper pathogenic differentiation and subsequent host invasion.

## Materials and methods

### Fungal culture

Wild-type (WT) strain B157 of *M. oryzae*, obtained from the Indian Institute of Rice Research, Hyderabad, India, was used for the study. The fungus was grown and maintained on YEG medium (glucose 1 g, yeast extract 0.2 g, H_2_O to 100 ml), oatmeal agar (Hi-Media), complete medium (CM) or prune agar (PA; Patkar et al., 2012). The spores were harvested from the biomass grown on PA medium. Photomorphogenesis or asexual development assays were done with fungal cultures grown on PA in the dark at 28°C for 3 days and then under constant illumination at room temperature for further 5 days to induce conidiation.

### Plasmid constructs and fungal transformation

The full-length *YPD1* gene (MGG_07173) was isolated from the B157 strain using gene-specific primers (YPD1F and YPD1R; Supplementary Table 1), and verified by sequencing. A stretch of 525 bp, which covers the second and third exon from the ORF of 1,558 bp, was amplified using the primers (YPD1 OF and YPD1 OR; Supplementary Table 1), and was used for making an RNAi construct. This sequence was analyzed by si-FI software tool (http://labtools.ipk-gatersleben.de/) to predict any off-target effects of siRNA. The RNAi construct was prepared in the pSilent vector (Nakayashiki, 2005), to generate a hairpin loop when expressed in the fungus. The vector pSil-YPD1 was constructed by cloning 525 bp of the *YPD1* ORF in the sense and antisense orientations on either side of the cutinase intron in the pSilent vector, under the constitutive TrpC promoter and terminator from *Aspergillus* sp. The *Xba* I-cleaved fragment of the construct was then mobilized into pCAMBIA 1,300 at the *Xba* I site, to obtain the binary construct pSil-YPD1. The plasmid construct was confirmed by sequencing. The construct was used to generate RNAi transformants using *Agrobacterium tumefaciens* mediated transformation (ATMT). The *A. tumefaciens* strain LBA4404/pSB1 was first transformed with the binary construct pSil-YPD1 via tri-parental mating using an *E. coli* strain carrying the helper plasmid pRK2013 (Ditta et al., 1980). The transformed *A. tumefaciens* strain was then used to transform *M. oryzae* via ATMT as described (Mullins et al., 2001). Briefly, *M. oryzae* spores were collected from 8 to 10-day old oatmeal agar or PA plates, and concentration adjusted to 10^6^ spores/ml. The *A. tumefaciens* strain carrying the binary construct pSil-YPD1 construct was grown at 28°C overnight in Luria-Bertani broth. Overnight-grown *A. tumefaciens* cells were diluted (OD_600_ 0.15) and grown in the induction medium (IM) for another 6 h. Fungal spores were co-cultivated with *Agrobacterium* induced with 200 μM acetosyringone (Sigma-Aldrich). The *M. oryzae* transformants were selected on complete medium (CM) or basal medium (BM) supplemented with the appropriate selection agent such as hygromycin B (Sigma-Aldrich) to a final concentration of 200 μg/ml or BASTA (40 μg/ml). The transformants were maintained as monoconidial isolates, as described, to obtain pure cultures (Gupta and Chattoo, 2007).

### Total RNA extraction, cDNA synthesis, transcript variants, and quantitative real-time PCR (qRT-PCR)

Fungal biomass, grown in liquid medium, was frozen in liquid nitrogen. Total RNA was isolated using TRIZOL reagent (Invitrogen Life Technologies). The quality of isolated RNA was checked by electrophoresis on formaldehyde gels and quantified by UV spectrophotometry. Total RNA (5 μg) was used to synthesize the first strand cDNA using M-MuLV reverse transcriptase (New England Biolabs) and random hexamer or oligo dT in a 20 μl reaction system (New England Biolabs). The promoter sequence and transcription start sites (TSS) in the *YPD1* locus were predicted using “promoter 2.0 prediction” tool (http://www.cbs.dtu.dk/services/Promoter/). Oligonucleotide primers (T0YPD1F and T1YPD1F; Supplementary Table 1) were designed based on the two potential TSS identified by the aforementioned online tool. The *YPD1* transcript variants T0 and T1 were then isolated, cloned and sequenced as follows—WT *M. oryzae* was grown in duplicate in liquid CM in dark at 28°C for 72 h. At the end of 3-day incubation, one set of the culture was shifted under light for photo-illumination for 6 h. The other set was continued in dark for 6 h. Total RNA was extracted from these two cultures, and the cDNA was amplified using the primers T0YPD1F, T1YPD1F, and T1T0YPD1R (Supplementary Table 1). The amplified cDNA products were cloned at the *EcoR* V site in pBluescript KS^+^. The cloned cDNA fragments were then sequenced using the M13 primer.

Quantitative real-time RT-PCR (qRT-PCR), to examine the expression pattern of *YPD1*, was performed using total RNA from 10^2^ conidia/ml geminated and grown for 72 h in liquid CM. qRT-PCR was performed on ABI 7900HT (Applied Biosystems, USA) using SYBR Green I and the requisite primer sets (Supplementary Table 1) for *YPD1* and different HK genes. Levels of *ENVOY* and blue light harvesting protein (*BLH*) transcripts were analyzed using the TaqMan probes (Applied Biosystems).

### Pathogenicity assays

Detached leaf infection assay was carried out using leaf blades from the susceptible rice cultivar CO39, and conidial suspension of *M. oryzae* (1 × 10^5^/ml in 0.2% gelatin). The leaf blades were covered with sterile mira cloth soaked in the aforementioned conidial suspension, and incubated in a humid chamber at 20°C with light/dark cycles (light for 14 h at 25°C and dark for 10 h at 20°C) for 4–7 days until full symptoms became apparent. Rice leaf sheath assay was carried out as described (Patkar et al., 2015). Spores were inoculated on the tender leaf sheath, incubated for about 36 h and observed under the microscope for penetration and invasive hyphae.

### Spore count and appressorial assays

The colonies were covered with ~5 ml of sterile distilled water. The spore suspension was obtained by carefully scraping the colonies with a sterile loop, and transferred into sterile falcon tubes. The spore suspensions were vortexed for 10–15 s and filtered through a sterile miracloth, to remove most of the mycelia. The filtered spore suspensions were centrifuged at 13,000 rpm for 4 min, washed and re-suspended in appropriate amount of sterile water. The number of spores was counted using a haemocytometer, and was expressed as conidia per unit area of the colony (cm^2^) per unit volume of the final suspension (ml).

A 20 μl drop of the conidial suspension (~1 × 10^4^/ml) was spotted on hydrophobic cover slips and left in 80% humid environment at 25°C. Appressorial development (morphology and the frequency of appressorium formation) was analyzed at appropriate time points (16–24 h post inoculation—hpi). Efficiency of appressorium formation was measured in at least three different samples, using more than 100 conidia per assay.

### Raising polyclonal antibodies against Ypd1

*M. oryzae YPD1* cDNA (435 bp) was amplified (see Supplementary Table 1 for primers) and cloned at the N-terminus of a 6x His Tag in pET30(a) for bacterial expression. An *E. coli* strain BL21 was transformed with the construct and was treated with 1 mM IPTG to induce expression of *M. oryzae* Ypd1. The fungal Ypd1 produced by *E. coli* was purified by affinity chromatography using an Ni-NTA resin (Thermo Scientific), concentrated and used for immunization of rabbits for generation of polyclonal antibody. The serum was collected after initial immunization with 300 μg of purified Ypd1 and subsequent two booster doses. The polyclonal antibodies were purified using a Protein A column and the titre was checked by ELISA with purified Ypd1 as the antigen. Western blot analysis was performed to confirm the reactivity of the Anti-Ypd1 antibody against purified fungal Ypd1.

### Western blot analysis

Total protein, for the detection of Hog1 or Ypd1, was extracted from the mycelia of the WT or *YPD1* KD transformant *M. oryzae* grown in liquid CM for 72 h, followed by washing and transfer to fresh liquid CM supplemented with or without a stress element for 6 h. The protein samples were electrophoresed on a 15% SDS-polyacrylamide gel, followed by electro-transfer to a PVDF membrane (Hybond-P; GE Healthcare lifesciences). Hybridization with the Ypd1 antibody was studied using a horseradish peroxidase-conjugated secondary antibody followed by detection with SuperSignal West Pico Chemiluminescence substrate (Thermo Scientific). Phosphorylation of Hog1 was studied using anti-phospho p38 MAPK antibody from Peirce Antibodies (Thermo Scientific). An anti-Hog1 antibody (Santa Cruz Biotechnology) was used to detect Hog1 expression as an internal control.

### Indirect immunolocalization and microscopy

DAPI (Sigma-Aldrich) staining for nuclei was performed as per the manufacturer's instructions. The immunolocalization of Ypd1 was performed using the Ypd1 antibody (1:200), at different stages of fungal development. The samples (conidiophore, mature conidia and germinating conidia) were fixed with 3.7% formaldehyde for 30 min at room temperature, followed by permeabilization using 0.1% Triton X-100. The fixed samples were further treated as described (Patkar and Chattoo, 2006), using anti-Ypd1 primary antibody and corresponding TRITC-conjugated secondary antibody. The samples were observed under an 100x objective on an epifluorescence (BX51; Olympus) or a 60x objective on a Zeiss LSM 700 confocal microscope. Images were processed using ImageJ or Adobe Photoshop CS2.

### Statistical analyses

Data involving analysis of total conidiation, appressorial development, and relative transcript levels of *YPD1* in the WT or *YPD1* knock-down (KD) transformants were statistically evaluated by the one-way ANOVA using Tukey's multiple comparison test. Relative transcript levels of *ENVOY, BLH* and various HKs in the WT or *YPD1* KD transformants were analyzed by the two-way ANOVA using the Bonferroni method.

## Results

### *M. oryzae* expresses two isoforms of *YPD1* in response to light

**Figure 1:**
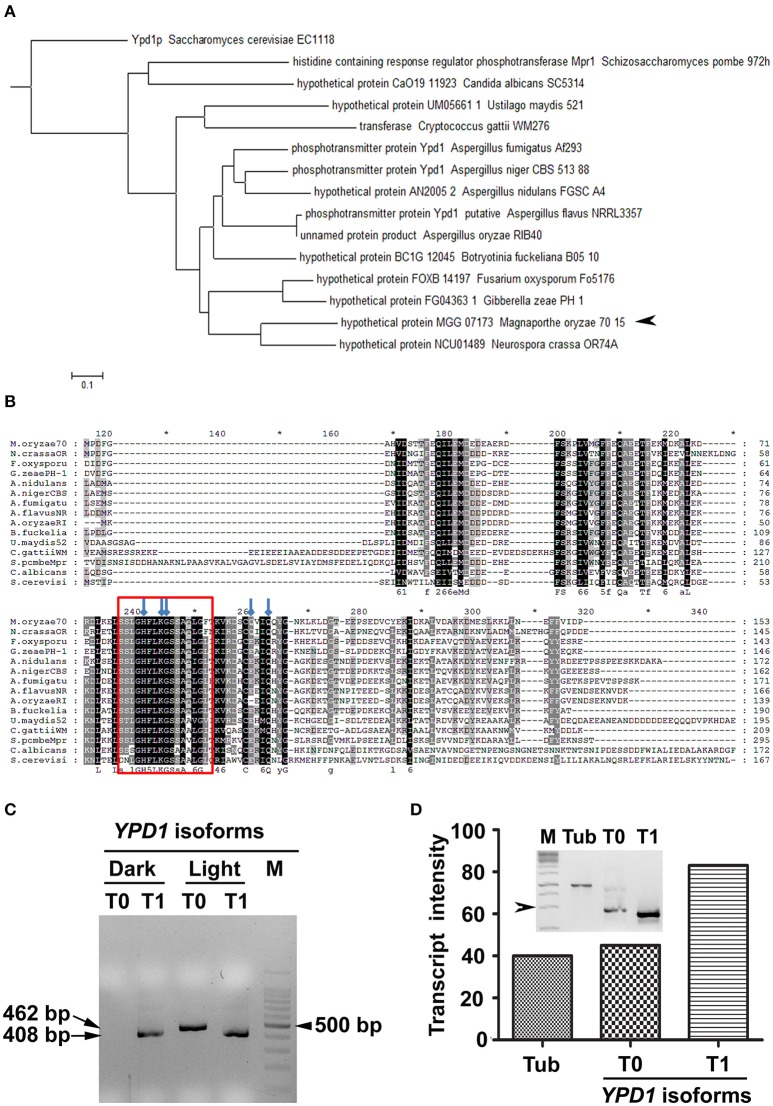
**Identification of Ypd1 and its gene isoforms in ***M. oryzae***. (A)** A dendrogram showing phylogenetic analysis of the Ypd1 sequences from various filamentous and non-filamentous fungi. Arrowhead denotes Ypd1 from *M. oryzae*. **(B)** Multiple sequence alignment of Ypd1 sequences from different fungi. The red box marks the highly conserved catalytic region in the Ypd1 sequence. Blue arrows show amino acids (H83, K86, G87, Q102, and Q105) crucial for functional folding of the protein. **(C)** Amplified cDNA products of the differentially accumulated *YPD1* isoforms (T0 or T1) under different growth conditions (dark vs. light). Size of the cDNA or a fragment from the 100 bp DNA ladder (M) is mentioned. **(D)** Graphical and qualitative (inset) representation of the ratiometric analysis of the transcript levels of the *YPD1* isoforms (T0 and T1) under photo-illumination, with respect to that of tubulin (Tub) as an internal control, in the WT *M. oryzae*. Arrowhead depicts 500 bp fragment from the 1 Kb DNA ladder (M). The asterisks denote 10-digit interval in the multiple sequence alignment.

We identified an ortholog of *S. cerevisiae YPD1* in the rice-blast fungus (MGG_07173) *M. oryzae*. Although the sequence homology to the *S. cerevisiae* Ypd1 was relatively low (44%), the *M. oryzae* counterpart had the conserved HPt domain with 57% sequence identity. Further, *in silico* analysis showed that *M. oryzae YPD1* had a 1,558 bp-long nucleotide sequence containing two introns, and coded for a protein with 161 amino acid residues. Overall, sequence similarity between *M. oryzae* Ypd1 and that from yeast and fungi ranged from 44 to 84% (Figures 1A,B). Importantly, the essential residues such as histidine (H83) required for phosphorylation and lysine (K86), glycine (G87), and glutamine (Q102 and Q105) involved in functional folding of HPt in *S. cerevisiae*, were conserved in *M. oryzae* Ypd1 (arrows; Figure 1B).

The genome annotation had predicted two transcript variants for *YPD1* in *M. oryzae* (https://www.broadinstitute.org/scientific-community/science/projects/fungal-genome-initiative/magnaporthe-comparative-genomics-proj). A previous study involving deletion of *YPD1* in *M. oryzae* showed that although the protein function was not required for viability, it was essential for conidiation (Jacob et al., 2015). Given that photo-illumination is crucial for conidiation or asexual development in *M. oryzae*, we studied by RT-PCR expression of *YPD1* under both dark and light conditions. Interestingly, we found an additional isoform of *YPD1*, likely resulting from the use of an alternative TSS upon exposure to light, in *M. oryzae*. The shorter *YPD1* transcript variant (T1; 408 bp) was expressed in the *M. oryzae* vegetative cultures grown in dark or light, whereas the longer isoform of the gene (T0; 462 bp) was found only in the fungal culture placed under photo-illumination (Figure 1C). Ratiometric analysis of the two isoforms of *YPD1* showed that the T1 transcript was relatively more abundant than the T0 in the WT grown under photo-illumination (Figure 1D). Further, we amplified, cloned and sequenced these two *YPD1* isoforms (see Section Materials and Methods), to study the nucleotide and amino acid sequences of the differentially expressed transcripts in *M. oryzae*. The additional 54 bp stretch in the N-terminus of the T0 isoform (Supplementary Figure 1A), coding for 18 amino acids, was analyzed for the putative phosphorylation sites using NetphosK (Blom et al., 1999). A potential casein kinase phosphorylation site was identified in the additional 18 amino acid stretch in the T0 isoform in *M. oryzae* (Supplementary Figure 1B). Thus, expression of two individual transcript variants under different growth conditions, suggests distinct functions for the two *YPD1* isoforms in *M. oryzae*.

### Indirect immunolocalization of Ypd1 in *M. oryzae*

**Figure 2:**
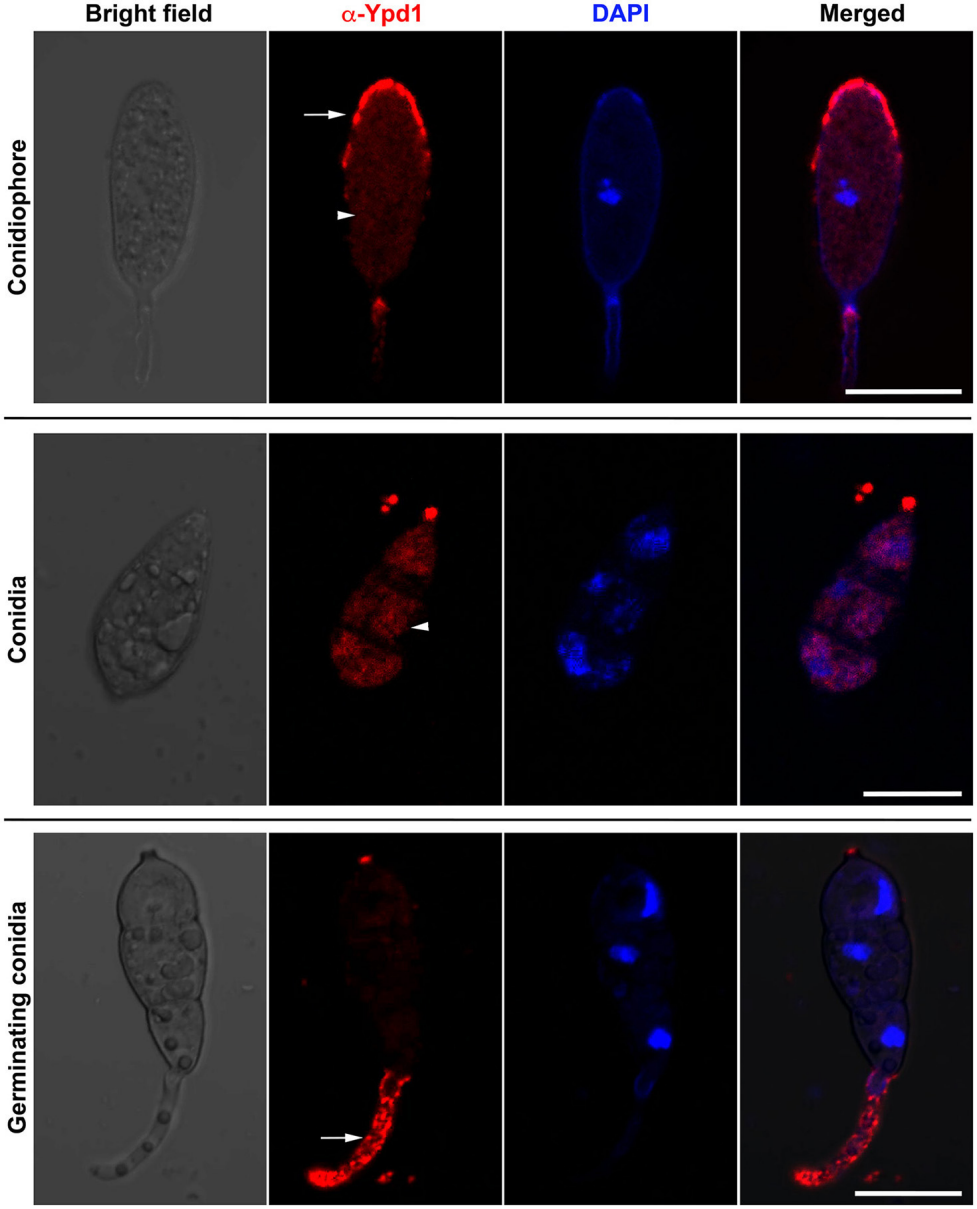
**Immunolocalization of Ypd1 in ***M. oryzae*****. Immunostaining of the fungal samples representing asexual development (conidiophores, upper panels; and mature conidia, middle panels) or pathogenic differentiation (germinating conidia, lower panels). Samples were co-stained with DAPI to mark the nuclei, and anti-Ypd1 antibody followed by TRITC-labeled secondary antibody. Arrowheads denote cytoplasmic signal whereas arrows show punctate signal. Scale bar, 10 μm.

We studied sub-cellular localization of Ypd1 in *M. oryzae* indirectly by immunolocalization using anti-Ypd1 primary antibody and TRITC-conjugated secondary antibody. The samples from various stages of fungal development, such as conidiophore, mature conidia, and germinating conidia, were co-stained with DAPI to aid visualization of the nuclei. The Ypd1 protein was detected in the cytoplasm of the developing, mature, and germinating conidia (white arrow heads; Figure 2). In addition, punctate aggregates of Ypd1 were seen at the tips of both the conidiophore and germ tubes (white arrows; Figure 2). We infer that Ypd1 likely localizes to the cytoplasm during asexual development in *M. oryzae*.

### Expression of light-inducible genes is dependent on Ypd1 in *M. oryzae*

**Figure 3:**
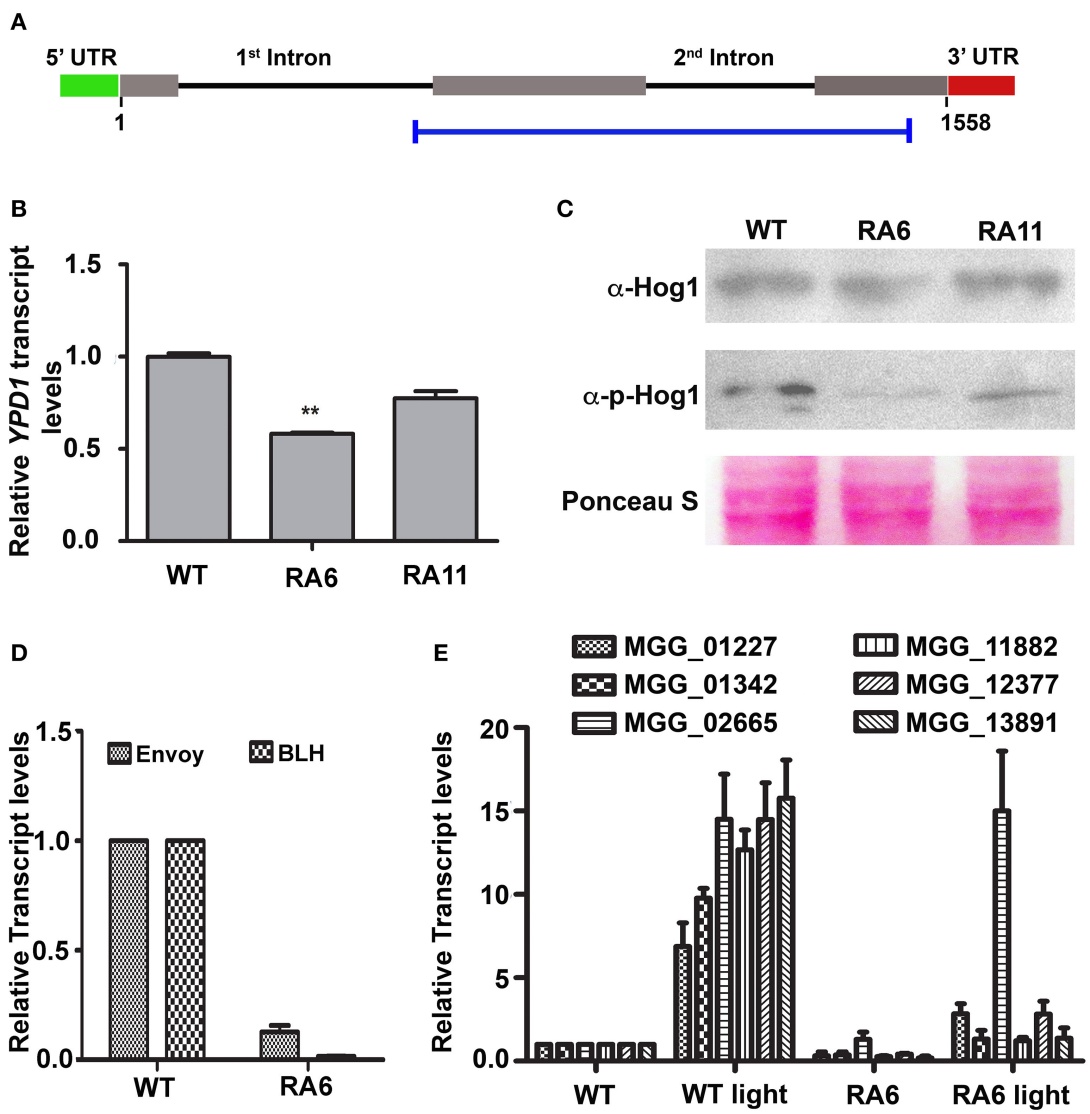
**Down-regulation of ***YPD1*** and its downstream effects in ***M. oryzae***. (A)** A schematic representation of the genomic locus of the *YPD1* open reading frame. The horizontal blue bar denotes the stretch of nucleotide sequence used to make a *YPD1* KD construct. **(B)** A bar graph representing relative *YPD1* transcript levels in the indicated KD transformants. Data represent mean ± s.e.m. from three independent experiments. ^**^P < 0.01. **(C)** Western blot analyses showing relative expression level of unphosphorylated or phosphorylated Hog1 protein detected using the relevant indicated antibodies. The blots were stained with Ponceau S to confirm normalization of protein samples. **(D)** Relative transcript levels of the indicated genes, between the WT or KD transformant RA6, are depicted in a bar graph. Data represent mean ± s.e.m. from three independent assays performed using total RNA from vegetative cultures of the indicated strains of *M. oryzae*. **(E)** A bar graph showing relative transcript levels of the indicated PAS-domain HKs in the WT or RA6 KD transformant grown under light or dark condition. Data represents mean ± s.e.m. from three individual experiments.

We transformed WT strain B157 to generate *YPD1* KD transformant (Figure 3A), to study function of the HPt in *M. oryzae*. Among 123 transformants obtained, 15 showed reduction in *YPD1* expression by 25–60% when compared to the WT. Transformants with more than 40% silencing could not survive further monoconidial isolation and repeated subculturing on the hygromycin-containing selection medium. We chose two of the KD transformants, RA6 and RA11 with ~40 and 25% silencing, respectively (Figure 3B; *P* < 0.005), for further studies. To study the role of Ypd1 in *M. oryzae*, we determined expression of the Hog1 MAPK in RA6 and RA11 mutants. Western blot analysis showed that while the overall expression of Hog1 was largely unaffected, phosphorylation of the MAPK was significantly reduced upon down-regulation of *YPD1* in both RA6 and RA11 KD transformants in *M. oryzae* (Figure 3C). This indicates that the Hog1 MAPK is likely a downstream target of Ypd1 in *M. oryzae*.

We further tested expression of light-regulated genes such as those coding for BLH protein (MGG_03002) or the PAS/LOV domain containing Envoy protein (MGG_01041). We found that both the genes were significantly down regulated in the *YPD1* KD transformant RA6 when compared to the WT (Figure 3D; *P* < 0.001). Next, we studied expression of the PAS domain HKs in response to light in *M. oryzae*. Exposure to light significantly induced expression of all the PAS domain HKs by about 6- to 15-fold in the WT (Figure 3E; *P* < 0.001). However, importantly, when *YPD1* was down-regulated, none of the HKs, except for MGG_02665, showed significant up-regulation even upon photo-illumination (Figure 3E; *P* < 0.001). It suggests that, except for MGG_02665, the light-inducible expression of PAS-domain HKs is dependent on Ypd1 in *M. oryzae*. Taken together, these results indicate that the fungal Ypd1 likely controls expression and activation of various PAS-domain HKs and MAPKs, respectively, to regulate diverse light-responsive functions in *M. oryzae*.

### Down-regulation of *YPD1* affects asexual development and pathogenesis in *M. oryzae*

**Figure 4:**
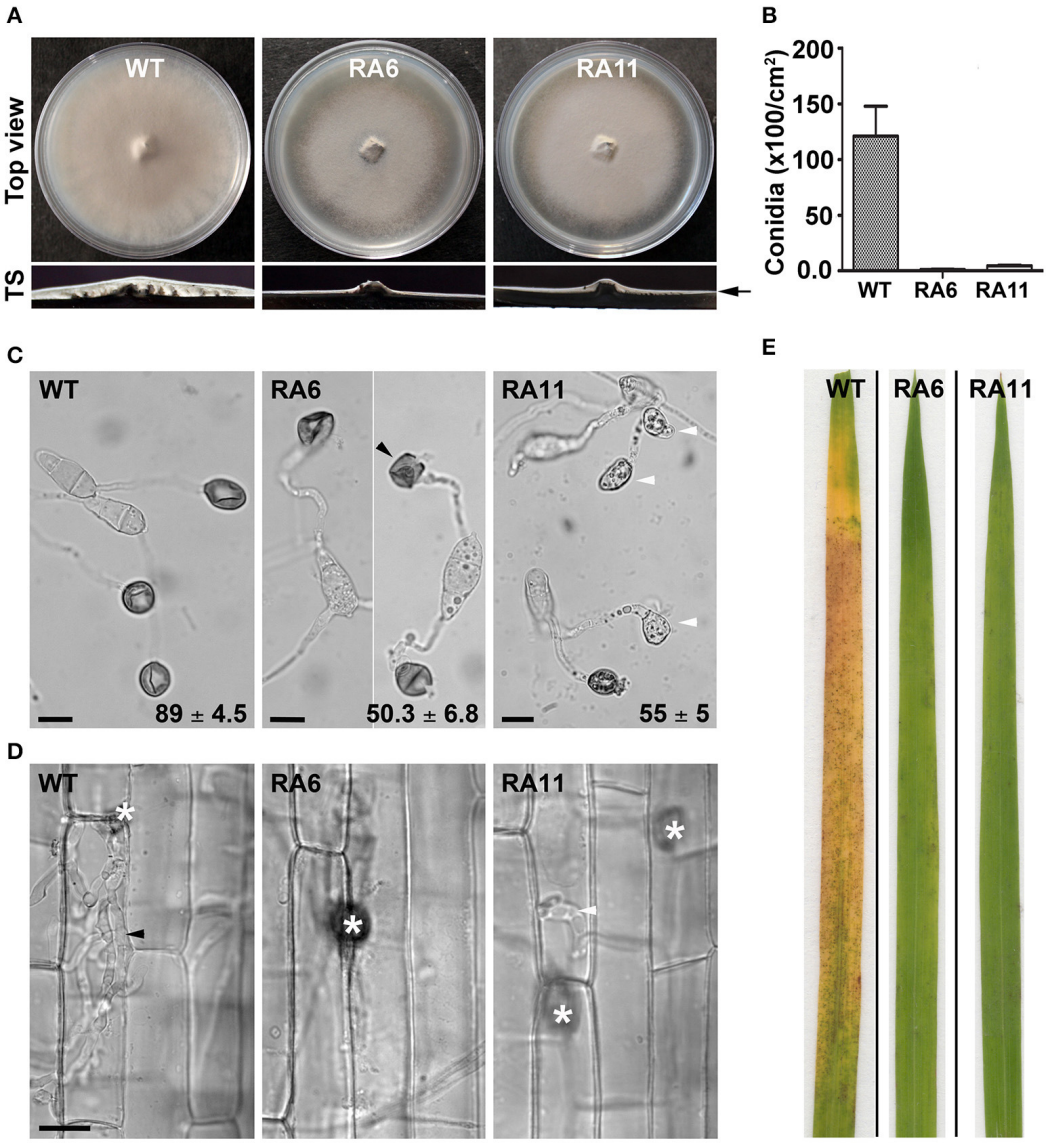
**Ypd1 plays a crucial role in asexual development and pathogenesis in ***M. oryzae***. (A)** Top panels show top view of the radial vegetative growth of the indicated *M. oryzae* strains after 10 days on PA medium. Transverse sections (TS) of the vegetative colonies showing aerial hyphal growth in the WT or KD transformants. Arrow depicts fluffy or thin aerial growth in the WT or the KD transformants, respectively. **(B)** A bar graph depicting total conidiation in the indicated strains of *M. oryzae*. Total conidia were harvested from 10-day old cultures of the strains grown on PA medium. Data represents mean ± s.e.m. from three independent experiments. **(C)** Micrographs showing appressorial development, on an inductive hydrophobic surface 24 hpi, by the indicated strain. Black and white arrowheads depict aberrant-shaped and non-melanized appressoria, respectively. Values indicate percentage (mean ± s.e.m. from three replicates) of appressorial development after 24 hpi. Scale bar, 10 μm. **(D)** Rice sheath was inoculated with the indicated strain of *M. oryzae*, and observed under bright field optics after 48 hpi. Asterisk shows appressoria on the sheath tissue surface. Black and white arrowheads depict elaborated or restricted fungal invasive hyphae, respectively, of the WT or *YPD1* KD transformant in *M. oryzae*. Scale bar, 10 μm. **(E)** Detached rice leaf blades were inoculated with the WT or the *YPD1* KD transformant conidial suspension, and the disease symptoms were assessed and photographed 7–8 days post inoculation.

Both the KD transformants RA6 and RA11 showed radial vegetative growth comparable to that of the WT on PA medium (top view; Figure 4A). However, unlike the WT that showed fluffy aerial growth, both the KD transformants formed rather flat colonies on PA medium (transverse section; Figure 4A). Total conidiation was highly reduced in the KD transformants RA6 and RA11 when compared to the WT (Figure 4B). The KD transformants RA6 and RA11 formed only 1.1 × 10^2^ and 4.5 × 10^2^ spores, respectively, as compared to 1.2 × 10^4^ conidia produced by the WT (Figure 4B; *P* < 0.005). We further assayed infection-related morphogenesis (appressorial development) and the ability to invade plant host, in the KD transformants, to study role of *YPD1* in *M. oryzae*. In an *in vitro* assay, while 89 ± 4.5% of the WT conidia formed appressoria on an inductive surface, only 50 ± 6.8 and 55 ± 5% of the RA6 and RA11 conidia, respectively, could develop appressoria (Figure 4C; *P* < 0.0005). In the KD transformants, appressoria were aberrant in morphology and melanization, when compared to the WT (Figure 4C). Most of these KD transformant appressoria failed to penetrate the host cuticle in rice leaf sheath inoculation assay (Figure 4D). The few KD transformant appressoria that did penetrate occasionally failed to elaborate the invasive hyphae and colonize the sheath tissue, when compared to the WT (Figure 4D). Thus, neither RA6 nor RA11 KD transformant could develop any obvious disease lesions on rice leaves, in the plant infection assay (Figure 4E). These results indicate that the failure of proper pathogenic differentiation and appressorial function (host invasion) was likely due to impaired signaling in a pathway downstream of Ypd1 in *M. oryzae*. Thus, we conclude that Ypd1 function is required for proper asexual development (photomorphogenesis) in the rice-blast fungus *M. oryzae*, likely via regulation of light-responsive gene expression and pathogenesis.

## Discussion

We characterized *M. oryzae* Ypd1 to study the role of HPt-mediated TCST in fungal development and pathogenesis. The catalytic site in the HPt domain in *S. cerevisiae* Ypd1 is marked with amino acid residues such as histidine, lysine, glycine, and glutamine, which are crucial for proper folding of the HPt toward efficient and stable phosphotransfer (Janiak-Spens and West, 2000; Porter and West, 2005).

Multiple sequence alignment showed a significantly conserved HPt domain containing the aforementioned residues, suggesting an evolutionary relatedness between the *M. oryzae* Ypd1 and the HPts from other fungi. The *M. oryzae* Ypd1 had the lowest or highest identity (44% or 84%) with *S. cerevisiae* Ypd1 or *N. crassa* HPt1, respectively. While Nc*HPT1* is essential for viability in *N. crassa*, the nc*hpt1*Δ mutant survived upon loss of a downstream Hog1 pathway protein Os-2 (Banno et al., 2007). Despite repeated attempts we failed to obtain a *YPD1*-deletion mutant in *M. oryzae*. In a previous study, although a *YPD1*-deletion strain was obtained, the Δ*ypd1* mutant showed a complete loss of conidiation in *M. oryzae* (Jacob et al., 2015). These suggest that Ypd1 function is essential for asexual reproduction in the rice-blast fungal pathogen. Therefore, we took the gene-silencing approach to generate a KD transformant and to study the role of the HPt during asexual and pathogenic development in *M. oryzae*. It remains to be investigated if loss/suppression of a downstream target protein function would rescue the defect in asexual development in the Δ*ypd1* mutant in *M. oryzae*. Ypd1 has been found to translocate between the nucleus and cytoplasm in *S. cerevisiae* and *C. albicans* (Lu et al., 2003; Mavrianos et al., 2014). However, sub-cellular localization of HPt has not been studied in filamentous fungi thus far. Our indirect immunolocalization study suggests that *M. oryzae* Ypd1 localizes uniformaly to the cytoplasm, and in the form of punctae at the growing tip of the conidiophore or germ tube during asexual or pathogenic development, respectively. A strain expressing Ypd1 tagged with a fluorescent marker is required to monitor the spatio-temporal expression and dynamics of the HPt in *M. oryzae*.

*M. oryzae* differentially expressed a longer transcript of *YPD1* when grown under constant photo-illumination. The N-terminal stretch of the longer transcript revealed a sequence which appears to be unique to *M. oryzae YPD1*, not having been found in any other fungal *HPT*s described so far. Further, an *in silico* analysis showed three potential phosphorylation sites including that for a casein kinase. Casein kinases function as regulators of signal transduction pathways and are involved in various processes including circadian rhythm and shuttling of transcription factors (Eide and Virshup, 2001). Interestingly, WCC, a transcription factor and a member of the circadian clock, binds to the promoter of the downstream target protein Os-4, in response to light, in *N. crassa* (Lamb et al., 2011). Further, the rhythmic expression of Hpt1 is indirectly regulated by the circadian clock in *N. crassa* (Lamb et al., 2011). Our findings suggest that Ypd1 likely functions during light response and/or circadian rhythm, possibly via differential expression of the gene isoforms, in *M. oryzae*.

Conidiation or asexual development is regulated by light in fungi. Constant photo-illumination is shown to induce conidiation in *Trichoderma reesei, A. nidulans, N. crassa*, and *M. oryzae* (Mooney and Yager, 1990; Olmedo et al., 2010; Chen et al., 2012). Such light-induced conidiation has been shown to be dependent on the activation of the blue light responsive WCC protein in *N. crassa* (Olmedo et al., 2010). Although, HKs and RRs from TCST pathways have been shown to be involved in asexual development, role of HPts in conidiation has been less explored in fungi. The *YPD1* KD transformants in this study showed a drastic decrease in conidiation in *M. oryzae*. Further, we found that the light-induced expression of ENVOY, BLH and PAS-domain HKs was dependent on Ypd1 in *M. oryzae*. Blue light is also known to control other cellular processes such as cell wall remodeling, mating, carbon metabolism in fungi like *N. crassa* and *T. reesei* (Schmoll et al., 2010, 2012; Gruber and Seidl-Seiboth, 2012). It is likely that Ypd1-mediated regulation of light-responsive genes could be involved in diverse functions in *M. oryzae*.

Our *in vitro* assays indicated that Ypd1 is likely involved in stress response in *M. oryzae* (Supplementary Figure 2). Previous studies have shown that the HKs (Hik1 and Sln1), HPt (through deletion of *YPD1*), and RRs (Ssk1 and Skn1) are likely involved in osmoresponse, hyphal melanization, resistance to fungicide and virulence in *M. oryzae* (Motoyama et al., 2008; Zhang et al., 2010; Jacob et al., 2014, 2015). The *YPD1* KD transformant studied here showed enhanced sensitivity toward osmotic stress and significantly reduced pathogenesis, similar to that seen in case of the aforementioned HK and/or RR mutants, suggesting their involvement in a common pathway(s) in *M. oryzae*. Although the Δ*ypd1* strain was found to be impaired in infectivity (Jacob et al., 2015), a complete loss of conidiation in this mutant made it difficult to study pre-invasive pathogenic differentiation (appressorial development) in the absence of the HPt function in *M. oryzae*. Importantly, our findings here indicate that the Ypd1 function is not required for appressorial development in *M. oryzae*. Given that the host penetration is marked with an oxidative burst (Huang et al., 2011) and that Ypd1 is likely involved in response to oxidative stress in *M. oryzae* (Supplementary Figure 2), we inferred that the reduction in pathogenesis in the *YPD1* KD transformants was most likely due to impaired appressorial function during host invasion. It would be worth testing if exogenous addition of an anti-oxidant would rescue the *YPD1* KD transformant defect in host invasion and colonization.

Overall, the involvement of *YPD1* in asexual development, pathogenic differentiation, and function in *M. oryzae*, suggests that the HPt could be a suitable target for an antifungal strategy in controlling rice-blast disease.

## Author contributions

Conceived and designed the experiments: BC, VM, and RP. Performed the experiments: VM. Analyzed the data: BC, JM, VM, PC, and RP. Contributed reagents/materials/analysis tools: BC. Wrote the paper: VM, JM, BC, and RP.

### Conflict of interest statement

The authors declare that the research was conducted in the absence of any commercial or financial relationships that could be construed as a potential conflict of interest.
