# Supplementary material for: Fungal Histidine Phosphotransferase Plays a Crucial Role in Photomorphogenesis and Pathogenesis in Magnaporthe oryzae
Source: Front Chem. 2017 May 19;5:31. doi: 10.3389/fchem.2017.00031 (PMC5437211; doi:10.3389/fchem.2017.00031)

Supplementary Information:

Supplementary Table S1: List of oligonucleotide primers used in this study.

Supplementary Figure 1: Light-induced *YPD1* isoform in *M. oryzae*. A) Nucleotide sequences of the *YPD1* cDNAs obtained from *M. oryzae* culture grown under photo-illumination. The extra stretch of nucleotide at the N-terminus of the T0 isoform is underlined. **B)** Phosphorylation sites predicted in the *YPD1* sequences in *M. oryzae*. Asterisks show additional potential phosphorylation sites in the T0 isoform when compared to those in the T1 variant.

Supplementary Figure 2: Ypd1 plays an important role in stress response in *M. oryzae*. A) Western blot analysis showing expression of Ypd1 under indicated stress condition (left panels). Total protein was extracted from *M. oryzae* cultures grown in liquid CM for 3 days followed by a treatment with or without the indicated compound for 6 h. Purified *E. coli*-produced recombinant Ypd1 was used as a positive control ((+) ve; right panels). Ypd1 was detected using the anti-Ypd1 antibody. The blot was stained with Ponceau S to confirm normalization of protein samples. **B)** Sensitivity of the indicated *M. oryzae* strains towards different stress conditions. Vegetative growth of the WT or YPD1 KD mutants grown on YEG with or without the indicated compound was assessed and photographed after 8 dpi.

Supplementary Table S1:

Name of the primer	Sequence of the primer
YPD1F	5’ ATGGTACCGTACGGTTGTTTGATGTCGG 3’
YPD1R	5’ ATGGATCCAGCCTGTCTTTCGTTTGAAG 3’
YPD1 OF	5’ATGGGCCCTAAGCTTGTATTGCCTAGAGCAGTCCGA 3’
YPD1 OR	5’ ACGGGCCCTAAGCTTGTCTCCATATCCTTCTTG 3’
YPD1 cDNA F	5’ CGACATATGCAGTCCGATTCCGAAGAAAAC 3’
YPD1 cDNA R	5’ CTCTCGAGAAGATCGATCACGAAAAACTCATTGAG 3’
T0YPD1F	5' ATGTACTCTGTAAAGGACTC 3'
T1YPD1F	5' ATGCCCGATTTTGGAGCCCA 3'
T1T0YPD1R	5' CTAAGGATCGATCACGAAAA 3'
YPD1q F	5’ AAGGGTTCATCCGCCACT 3’
YPD1qR	5’ ATAGCACACATCCTCGCTTG 3’
TUBqF	5’ GAGTCCAACATGAACGATCT 3’
TUBqR	5’ GTACTCCTCTTCCTCCTCGT 3’
MGG_01342qF	5’ TTTGGCCGGCAAGTCAACGGAA 3’
MGG_01342qR	5’ AATCCCTCGGCGGTTTTCAGCT 3’
MGG_13891qF	5’ ACTTCGCAAAGCCGGATGCA 3’
MGG_13891qR	5’ ATTACCGACAGCCGCACGTT 3’
MGG_11882qF	5’ ACATTCGCTTGCCCACCTTGCA 3’
MGG_11882qR	5’ AAATCGGGGTTGCGCTTCTGGA 3’
MGG_12377qF	5’ AGCGCAAAGGCATTGGCTACGA 3’
MGG_12377qR	5’ TGCGATGTGTGTTGGACAGCGT 3’
MGG_01227qF	5’ ATTTGAACGGAGCATCGCCGCA 3’
MGG_01227qR	5’ TTGTTGCCGCACCTGACAAGCT 3’
MGG_02665qF	5’ ACCAACAGGTCACGCGACGAAT 3’
MGG_02665qR	5’ TGCAATCCCGTTGTGGCTACTG 3’

Supplementary Figure S1:

A

***YPD1* isoform T1 (408 nt):**

ATGCCCGATTTTGGAGCCCATGTCGACAGCACGACGTTCGAACAGATCCTGGAAA
TGGACGAAGATGAGGCCGAAAGGGATTTCAGCAAACCTTTAGTCATGGGATTCTT
TGAACAAGCAGAGGAGACTTTTGAAAAAATGGACAAGGCCCTGAAAGATCGTGA
CTTGAAAGAGCTTTCGAGCCTTGGCCACTTCCTCAAGGGTTCATCCGCCACTCTG
GGCTTTACAAAGGTCAAGGACAGCTGCCAAGTGATTCAACAGTACGGAAACAAG
CTGAAACTAGACGGCACTGAGGAGCCAAGCGAGGATGTGTGCTATGAGAAGATC
GATAAAGCACTTGTGGATGCCAAGAAGGATATGGAGAGTTTGAAGAAACTTCTCA
ATGAGTTTTTCGTGATCGATCCTTAG

***YPD1* isoform T0 (462 nt):**

ATGTACTCTGTAAAGGACTCTGAAAAGTCCGATTCCGAAGAAAACGCCGACAAGATGCC
CGATTTTGGAGCCCATGTCGACAGCACGACGTTCGAACAGATCCTGGAAATGGACGAAG
ATGAGGCCGAAAGGGATTTCAGCAAACCTTTAGTCATGGGATTCTTTGAACAAGCAGAG
GAGACTTTTGAAAAAATGGACAAGGCCCTGAAAGATCGTGACTTGAAAGAGCTTTCGAG
CCTTGGCCACTTCCTCAAGGGTTCATCCGCCACTCTGGGCTTTACAAAGGTCAAGGACA
GCTGCCAAGTGATTCAACAGTACGGAAACAAGCTGAAACTAGACGGCACTGAGGAGCC
AAGCGAGGATGTGTGCTATGAGAAGATCGATAAAGCACTTGTGGATGCCAAGAAGGATA
TGGAGAGTTTGAAGAAACTTCTCAATGAGTTTTTCGTGATCGATCCTTAG

B

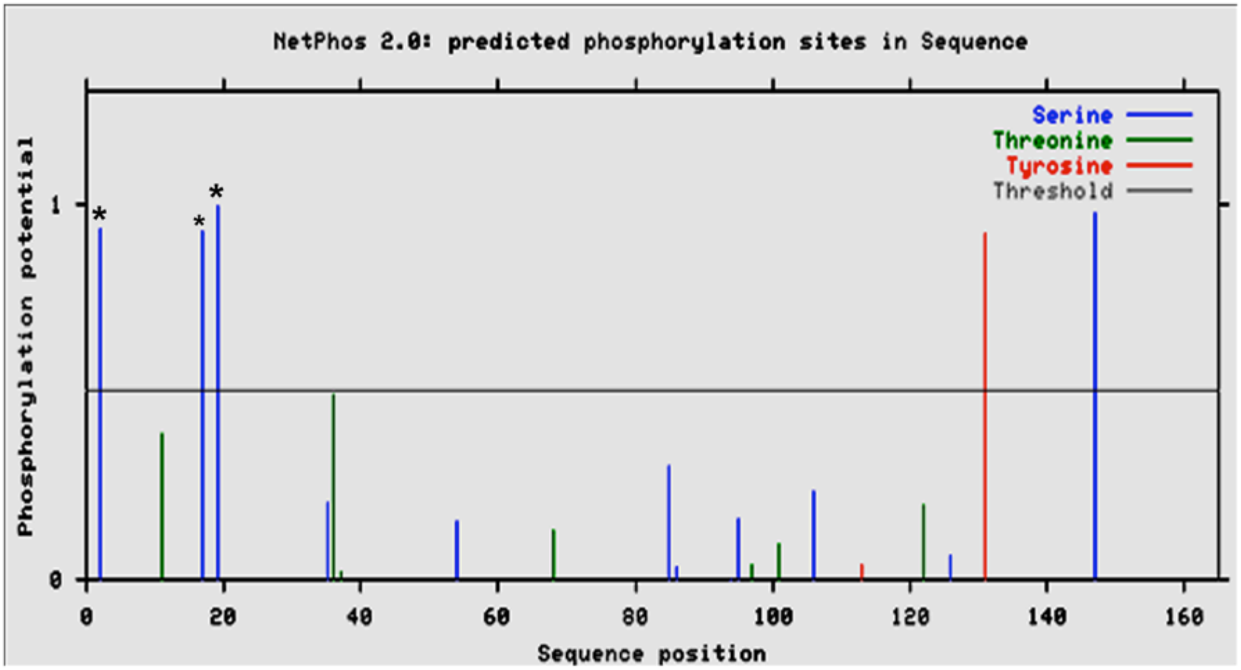

Supplementary Figure S2:

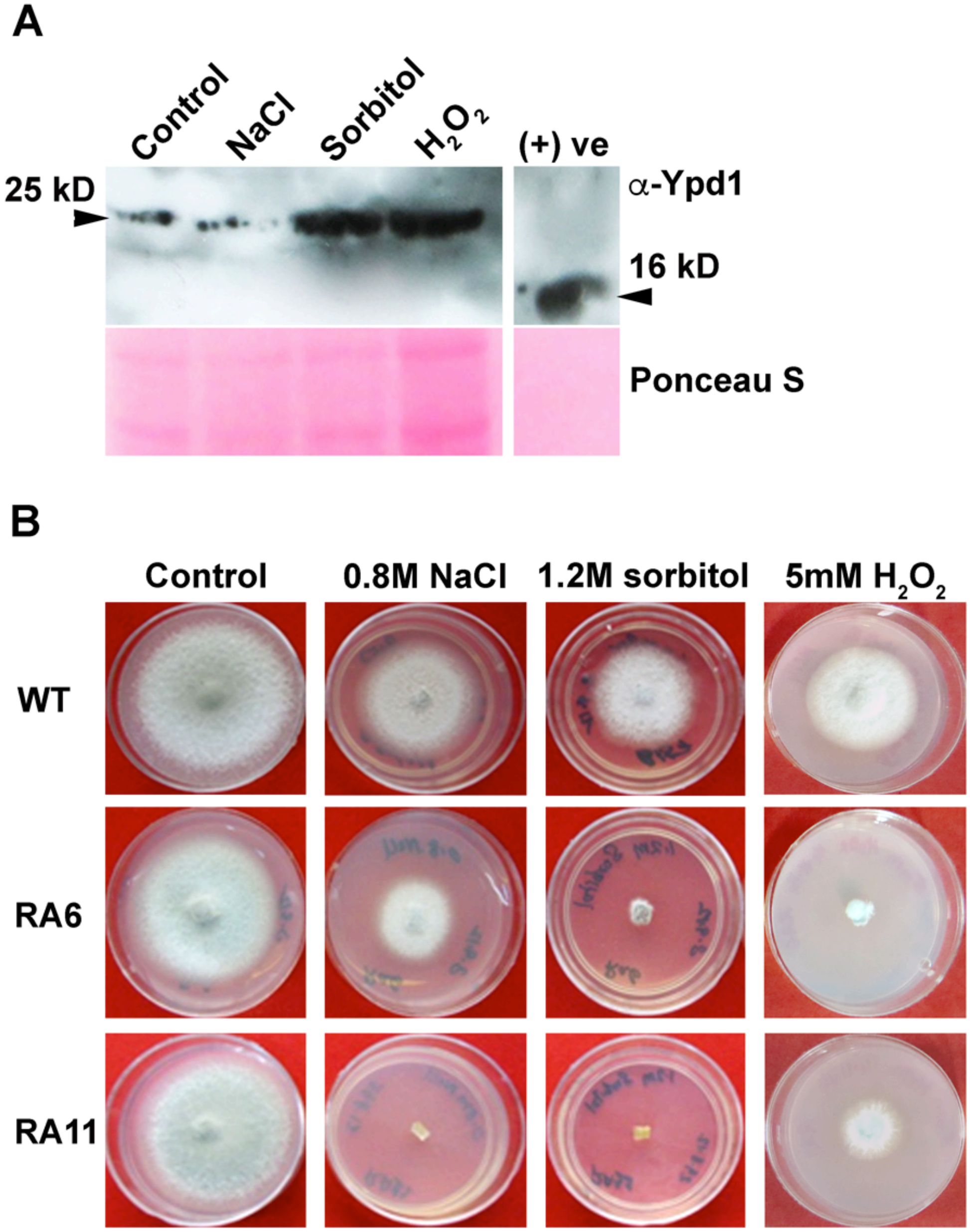

Supplement: Supplementary file 1 [file Presentation1.pdf]
